# Heart Rate Responses During Official Kick Light Competition and Their Associations with Ventilatory Thresholds and VO_2_max: An Exploratory Study

**DOI:** 10.3390/sports14090415

**Published:** 2026-09-20

**Authors:** Paulo Sousa-e-Silva, Guilherme Furtado, Gabriela Costa, Diogo Vicente Martinho, Ricardo Gomes

**Affiliations:** 1 Polytechnic University of Coimbra, Rua da Misericórdia, Lagar dos Cortiços, S. Martinho do Bispo, 3045-093 Coimbra, Portugal; guilherme.furtado@ipc.pt (G.F.); rimgomes@esec.pt (R.G.); 2 SPRINT—Sport Physical Activity and Health Research and INnovation cenTer, Polytechnic University of Coimbra, Rua Dom Joao III—Solum, 3030-329 Coimbra, Portugal; 3 Interdisciplinary Center for the Study of Human Performance (CIPER), Faculty of Sport Sciences and Physical Education, University of Coimbra, 3040-248 Coimbra, Portugal; diogomartinho@fcdef.uc.pt; 4 Faculty of Sport Sciences and Physical Education, University of Coimbra, 3040-248 Coimbra, Portugal; gabi2001costa@gmail.com

**Keywords:** ventilatory, thresholds, maximal aerobic power, physiological profile, combat sports, kickboxing

## Abstract

Kickboxing is a hybrid striking combat sport characterized by intermittent, acyclic activity that taxes both aerobic and anaerobic energy systems. Yet, physiological data from tatami disciplines such as Kick Light (KL) remain scarce. This exploratory study examined ventilatory thresholds and heart rate (HR) responses in a university kickboxing team comprising eight athletes (six males, two females) across 15 official bouts, focusing on intensity relative to the first and second ventilatory thresholds (VT1, VT2). One week before competition, athletes completed an incremental treadmill test to determine VT1, VT2, and maximum oxygen consumption (VO_2_max). During competition, HR was continuously monitored to quantify time spent below VT1, between VT1 and VT2, and above VT2, as well as maximal HR during rounds and HR recovery during breaks. VT1 and VT2 occurred at approximately 49% and 90% of VO_2_max, respectively, with VO_2_max averaging 52.46 ± 5.14 mL·kg^−1^·min^−1^. In competition, athletes spent most of Round 1 between VT1 and VT2, whereas Round 2 showed a shift towards intensities above VT2 (increase in 7 of 8 athletes; *p* = 0.018). Associations were examined at the athlete level *(n* = 8) after averaging repeated bout observations within each athlete. Higher VO_2_max was associated with greater time below VT1 in Round 1 and lower average in-competition HR. These exploratory findings provide preliminary information on cardiovascular responses during official university Kick Light competition and may inform future, larger studies.

## 1. Introduction

Kickboxing is a hybrid combat sport that emerged from the combination of striking disciplines such as Boxing, Karate, and Muay-Thai, and is characterized by intermittent, acyclic actions that rely on both aerobic and anaerobic energy systems [1]. In competitive settings, athletes repeatedly perform short bursts of high-intensity techniques followed by brief recovery periods, which creates substantial metabolic stress and demands both a well-developed aerobic base and the ability to recover quickly between rounds and, in tournament formats, between successive bouts on the same day [2,3].

The World Association of Kickboxing Organizations (WAKO) is the official international governing body for kickboxing within the Olympic movement [4]. This step consolidated kickboxing’s status as a globally regulated combat sport and underscored the need for discipline-specific physiological research to support evidence-based training prescription, competition monitoring, and injury-prevention strategies across different competitive contexts.

Under WAKO rules, kickboxing is divided into ring disciplines, in which full contact and knockouts are allowed, and tatami disciplines, which prioritize controlled contact, accurate scoring, and strategic movement. Kick Light (KL) is a tatami discipline that emphasizes technical accuracy, tactical use of distance, and well-controlled contact, but still requires athletes to sustain frequent high-intensity offensive and defensive actions. In single-day university tournaments, KL athletes typically contest two to three two-minute rounds with one-minute intervals, and may perform several fights in the same event, from qualifiers to the finals, which makes adequate aerobic fitness and efficient recovery between rounds and bouts particularly important. Additionally, poor conditioning may be associated with an increased risk of injury [1].

Although kickboxing has grown in popularity and gained Olympic recognition, the scientific literature on its physiological demands is still relatively limited, especially for tatami disciplines such as KL. Studies conducted in different countries have reported maximum oxygen consumption (VO_2_max) values in amateur, elite, and professional kickboxers, but have used a range of ergometers and protocols that make direct comparison difficult. For example, VO_2_max values of 51.9 ± 4.3 mL·kg^−1^·min^−1^ were reported in amateur Tunisian kickboxers [5]. In contrast, values above 62 mL·kg^−1^·min^−1^ were reported in elite professional Canadian and elite Portuguese kickboxers [6,7]. In the Tunisian and Canadian samples, VO_2_max was assessed with cycle-ergometer protocols, which may underestimate peak oxygen uptake in trained fighters compared with treadmill-based tests [6].

Beyond absolute VO_2_max values, the relationship between aerobic capacity and technical–tactical performance has also been explored. A study of 20 high-level kickboxers from Poland found a strong positive correlation between VO_2_max and participants’ activeness, effectiveness, and efficiency, suggesting that the athlete’s level of technical and tactical training is related to aerobic power [3]. However, most of this evidence relates to ring disciplines, and there is still a lack of data describing the physiological profile of tatami athletes, including KL competitors.

Ouergui et al. [8] analyzed the physiological responses of different Kickboxing styles (Full Contact, Light Contact, Point Fighting). After a simulated Light Contact competition, the 18 male kickboxers (regional and national level) exhibited higher blood lactate levels (15.8 ± 4.0 mmol/L), reached 87% of the maximum HR, and 77% of the time was spent in Z4 (80–89% of the maximum HR) and Z5 (90–100% of the maximum HR). It should be noted that KL was not included in Ouergui et al. study, although Light Contact is the style with more similarities to KL. Additionally, the use of equations that estimate HRmax [9,10], as well as the use of a typical five-zone model that is based on standardized zones [11], can lead to an incorrect exercise prescription [12,13,14].

Evidence from related striking sports reinforces the relevance of ventilatory thresholds for interpreting internal load. De Lira et al. [15] assessed VO_2_max and ventilatory thresholds in highly trained Olympic boxing athletes and used the heart rate associated with each threshold to categorize intensity during a simulated match. About 60% of the simulated boxing match occurred at an intensity above the second ventilatory threshold (VT2). However, these data should be interpreted with caution, as the duration of the competition was lower (three rounds of two minutes) than that of an official Olympic boxing competition (three rounds of three minutes), and official and simulated events differ considerably in physiological terms [16].

To the best of our knowledge, no previous study has investigated the cardiovascular responses of KL athletes during an official competition. Characterizing how KL athletes distribute effort across ventilatory-based intensity zones during real tournaments could provide more precise information on match demands and help coaches design training programs that better reflect the actual competitive load, moving beyond generalized heart rate zones towards individualized, physiologically grounded prescriptions. Therefore, the aim of this work is twofold: to determine the ventilatory thresholds and VO_2_max of KL university athletes and, based on this first objective, to explore how the cardiovascular system of these athletes responds during a real KL competition, identifying the intensity zones at which they compete. We hypothesized that competition would elicit heart rate responses in the moderate-to-vigorous intensity range, with mean values approaching or surpassing VT2, and that heart rate would remain elevated between rounds, indicating incomplete recovery.

## 2. Materials and Methods

### 2.1. Study Design

This study followed a cross-sectional design involving a university Kick Light (KL) team. One week before the national university KL championship, athletes completed a laboratory incremental treadmill test to determine VO_2_max and ventilatory thresholds (VT1, VT2). During the official competition, heart rate (HR) was continuously monitored to characterize cardiovascular responses and time spent in intensity zones defined by those thresholds.

### 2.2. Participants

Eight university kickboxers (6 male and 2 female) participated in this research. The participants were all enrolled in the national KL university tournament. The age and anthropometric characteristics are described in Table 1. These values refer to the participants’ characteristics in the week before the competition, when their body composition and cardiorespiratory fitness were assessed. Sample size was not determined by a formal a priori power analysis. All available athletes from the university Kick Light team who met the inclusion criteria and agreed to participate were included, resulting in a convenience sample of eight athletes. The study was therefore exploratory and descriptive in nature.

### 2.3. Anthropometry and Body Composition

Upon arrival at the laboratory, stature was measured using a portable stadiometer (SECA 213, SECA GmbH & Co. KG, Hamburg, Germany). The participants were instructed to stand barefoot and straight, to guarantee their alignment with the Frankfort plane. After this, their body composition was assessed with a multifrequency bioelectrical impedance analyzer (InBody 270, InBody Co., Ltd., Seoul, Republic of Korea), following the manufacturer’s standardized testing protocol (e.g., barefoot, fasted, and free of exercise or caffeine for at least 3 h before assessment). The analyzer provided total body mass and fat mass, which were later used for descriptive characterization of the sample.

### 2.4. Incremental Treadmill Test and Gas Analysis

VO_2_max and ventilatory thresholds were assessed with a continuous incremental treadmill protocol, on a motorized treadmill (LODE Valiant 2 Sport, Groningen, The Netherlands), set at a constant incline of 1% to better reflect the energy cost of level outdoor running [17].

After a 3 min warm-up at 7 km/h, the participants began the test at 8 km/h, with subsequent increments of 1 km/h per minute until volitional exhaustion. Before each test, participants were fitted with a nasal–oral mask connected to a portable gas analyser (MetaMax 3B-R2, Cortex Biophysik GmbH, Leipzig, Germany). The system was calibrated before each session following the manufacturer’s guidelines.

Ventilatory data were collected breath-by-breath and subsequently exported for analysis. To reduce random fluctuations and facilitate threshold detection, data were visually inspected to remove evident artefacts and then averaged into 30 s intervals. VO_2_max values and ventilatory transition points were obtained using MetaSoft Studio software 5.0 (Cortex Biophysik GmbH, Leipzig, Germany).

Heart rate was continuously recorded throughout the protocol using a chest-strap heart rate monitor (Polar H7, Polar Electro, Kempele, Finland). Ratings of perceived exertion (RPE) were acquired after the end of each stage using the CR-10 scale [18], which was previously explained to the participants in a brief familiarization session.

### 2.5. VO_2_max Criteria

VO_2_max was primarily defined by the presence of a VO_2_ plateau, operationalized as a change ≤ 2.1 mL·kg^−1^·min^−1^ between the two highest consecutive 30 s mean values of oxygen uptake [19]. As a complement, the effort was confirmed to be maximum through the concomitant fulfillment of the following secondary criteria: (1) RER ≥ 1.05; (2) ≥90% of the predicted HRmax [20]; (3) RPE between 8 and 10 on the CR-10 scale [18]. Although the use of predicted HRmax equations to define training intensity zones is discouraged due to large inter-individual variability, ≥90% of predicted HRmax was used here only as one of several secondary criteria to confirm that a high level of effort was achieved during the VO_2_max test. The following variables were retained for subsequent analysis: oxygen uptake (VO_2_), HR, RER, RPE, and treadmill speed corresponding to VT1, VT2 and VO_2_max.

### 2.6. Ventilatory Threshold Determination

Ventilatory thresholds were identified based on the visual and graphical analysis of the values of ventilated gases (O_2_ and CO_2_) by two independent researchers (A and B), one of them considered experienced (>200 tests; researcher A). Both investigators independently determined VT1 and VT2 for each athlete before any consensus procedure. For each athlete and each threshold, the VO_2_ value at the point identified by each researcher was recorded. When the difference between the two VO_2_ values was below 3% of the mean, the average of the two values was used as the final threshold for that athlete, in line with the procedure described by Gaskill et al. (2001) [21]. If the difference exceeded 3%, the researchers met and examined the data together to reach mutual agreement. For VT1, thresholds for 7 of the 8 athletes were within the predefined 3% agreement criterion and were accepted as the mean of the two values, whereas VT1 for 1 athlete required a consensus procedure. VT2 determinations for all 8 athletes met the 3% criterion and did not require consensus.

VT1 was determined primarily by the ventilatory equivalents method: VT1 was identified at the point where the ventilatory equivalent for oxygen (VE/VO_2_) began to rise without a corresponding increase in the ventilatory equivalent for carbon dioxide (VE/VCO_2_) [22,23]. As a complement, end-tidal oxygen pressure (PetO_2_) demonstrates its first rise [24], and there is also a break in the linearity between VO_2_ and VCO_2_ due to the exponential increase in VCO_2_ relative to VO_2_ (namely referred to as the v-slope method) [23,25].

VT2 was identified at the point where there is a disproportionate increase in VE concerning VCO_2_ [25], concomitantly with an increase in VE/VCO_2_ in the graph of the ventilatory equivalents. Consequently, there is a significant reduction in PetCO_2_ [24] since hyperventilation cannot compensate for excess H+. An additional qualitative marker for VT2 was the sharp increase in breathing frequency (BF), reflecting the transition to more pronounced hyperventilatory responses.

### 2.7. Competition Format and Heart Rate Monitoring

For the second phase of this study, participants competed in the national university KL championship. Matches consisted of two rounds of two minutes each, with a one-minute interval between rounds, except for the finals, which followed the traditional three-round format. Three of the 15 fights were finals. Therefore, only the Round 3 data from these three final bouts were excluded from the analysis, whereas the Round 1 and Round 2 data from all 15 bouts were included.

During combat, participants wore a Polar H7 chest-strap HR monitor (Polar Electro, Finland) directly against the skin beneath the sleeveless competition T-shirt. The monitor was positioned around the lower chest according to the manufacturer’s instructions and was not externally visible. Participants wore the mandatory Kick Light protective equipment, according to WAKO Kick Light Rules, including head guard, mouth guard, kickboxing gloves, hand wraps, groin protection, shin guards, and foot protection. Female participants also wore breast protection beneath the competition top. HR data were segmented into combat and rest phases for each fight, allowing the computation of: (1) time spent below VT1 (Zone 1, Z1); (2) time between VT1 and VT2 (Zone 2, Z2); (3) time above VT2 (Zone 3, Z3); (4) maximal HR during rounds; (5) heart rate recovery (ΔHR) between rounds, defined as the difference between final HR at the end of Round 1 and the minimum HR observed during the subsequent rest interval. Heart rate zones were calculated over the entire round duration, including non-fighting periods, to reflect the overall physiological load experienced by the athletes during the round.

### 2.8. Statistical Procedures

Descriptive statistics were used to characterize participants’ anthropometric data, cardiorespiratory values, and heart rate responses in competition. Central tendency and dispersion metrics (mean, median, standard deviation, interquartile range) were calculated for key variables.

Given the repeated-fight structure of the competition, the athlete was considered the independent statistical unit for correlational analysis. Repeated fight observations were first averaged within each athlete before calculating associations, resulting in one single observation per athlete (*n* = 8). Exploratory associations were examined using Spearman’s rank correlation coefficient (ρ). A correlation matrix was retained to descriptively characterize the observed athlete-level association patterns. Because Z1, Z2, and Z3 represent compositional percentages that sum to approximately 100%, correlations among HR-zone variables were considered descriptive and were not interpreted as independent physiological relationships. Inferential interpretation focused primarily on physiologically relevant associations involving VO_2_max, mean competition heart rate, and ΔHR.

Round-to-round changes in the proportion of time spent above VT2 (Z3) were examined at an athlete level. Repeated fight observations were averaged for each athlete in Round 1 and 2, and the resulting paired values were compared using the Wilcoxon signed-rank test. Linear mixed-effects models were fitted to evaluate changes in mean heart rate between rounds, while accounting for repeated observations within athletes and the uneven number of fights. Round was used as a fixed effect (coded 0 = Round 1, 1 = Round 2) and subject as a random intercept. The model was estimated using restricted maximum likelihood (REML) with the Kenward–Roger approximation for denominator degrees of freedom. Model assumptions were assessed through visual inspection of normal Q-Q plots and residual vs. fitted plots.

Given the small sample size, the analysis was considered exploratory. Model estimates are presented as beta coefficients (β) with 95% confidence intervals. Statistical significance was set at *p* < 0.05.

### 2.9. Ethical Considerations

The research was conducted in accordance with the principles of the Declaration of Helsinki (2013 revision) [26]. The research protocol was reviewed and approved by the Ethics Committee of the Polytechnic of Coimbra (approval number 112/2025). All participants were informed about the procedures, potential risks, and benefits of the study, and provided written informed consent before participation.

## 3. Results

### 3.1. Ventilatory Responses During the Treadmill Step Test

Table 2 presents the descriptive ventilatory responses of the athletes during the incremental test. At the first ventilatory threshold (VT1), athletes reached an average oxygen consumption of 1.82 L·min^−1^, corresponding to 26.59 ± 5.33 mL·kg^−1^·min^−1^ and approximately 49% of VO_2_max (52.46 ± 5.14 mL·kg^−1^·min^−1^), with mean heart rate values at 137 bpm and breathing frequency (BF) at 28.93 ± 7.14 breaths·min^−1^.

At the second ventilatory threshold (VT2), mean oxygen uptake increased to 3.35 ± 0.61 L·min^−1^ (46.81 ± 4.33 mL·kg^−1^·min^−1^), corresponding to 89.94 ± 6.33% of VO_2_max. Heart rate rose to 183.50 ± 11.33 bpm and breathing frequency to 44.34 ± 9.31 breaths·min^−1^, accompanied by RER values around 1.00 ± 0.04, indicating a clear shift towards heavy–severe intensity with marked ventilatory and metabolic stress.

At peak exercise (VO_2_max), athletes exhibited mean oxygen consumption of 3.74 ± 0.68 L·min^−1^ (52.46 ± 5.14 mL·kg^−1^·min^−1^), heart rate of 192.38 ± 8.81 bpm, BF of 55.44 ± 9.97 breaths·min^−1^, and RER of 1.08 ± 0.04. All eight athletes met the primary VO_2_ plateau criterion and complementary markers of maximal effort, supporting the interpretation of true maximal effort during the incremental protocol.

### 3.2. Heart Rate Responses During Official Competition

Table 3 describes the heart rate responses during official competition. For variables with highly skewed distributions (e.g., percentage of time in specific heart rate zones), medians and interquartile ranges are reported alongside means and standard deviations and should be prioritized when interpreting these data. In Round 1, the athletes spent most of the fighting time within Z2 (median 59.10%, IQR 13.10–96.00%; mean ± SD: 52.77 ± 37.72% of total round time between VT1 and VT2), with additional exposure to Z3 (above VT2; median 0.70%, IQR 0.00–86.90%; mean ± SD: 35.23 ± 42.21%) and comparatively less time in Z1 (below VT1; below VT1; median 0.00%, IQR 0.00–15.70%; mean ± SD: 12.03 ± 22.67%). Average HR was 178 ± 9 bpm, and final HR reached 185 ± 9 bpm.

In Round 2, the cardiovascular load was even more pronounced, with time in Z3 increasing (median 46.40%, IQR 27.25–98.50%; mean ± SD: 55.55 ± 37.32%), time in Z2 decreasing (median 37.75%, IQR 1.50–72.75%; mean ± SD: 40.91 ± 35.76%), and Z1 becoming almost negligible (median 0.00%, IQR 0.00–0.78%; mean ± SD: 3.54 ± 9.01%). Average heart rate approached 181.50 ± 8.65 bpm, and final heart rate rose to 189.14 ± 8.96 bpm, confirming the predominance of high-intensity physiological stress during the second round.

### 3.3. Correlation Analysis

Table 4 summarizes the athlete-level exploratory associations between ventilatory-based intensity distribution during competition, aerobic power (VO_2_max), and competition heart rate responses. This analysis aimed to determine whether the physiological profile obtained during laboratory testing was associated with the internal load responses recorded in competition. Repeated fight observations were first averaged within each athlete, so that each participant contributed to a single observation to each correlation (*n* = 8).

Higher aerobic power (VO_2_max) was strongly and positively associated with a greater mean proportion of Round 1 time spent in Z1 (R1 Z1: ρ = 0.913; *p* < 0.002) and negatively associated with mean competition heart rate during R1 (ρ = −0.719; *p* = 0.045).

Time spent in Z3 showed a negative, non-significant trend in its relationship with ΔHR. Smaller ΔHR tended to co-occur with a greater percentage of time in Z3 during Round 2 (R2 Z3 vs. ΔHR: ρ = −0.690; *p* = 0.058), but this trend did not reach conventional statistical significance and should be interpreted cautiously in this small exploratory sample. Athlete-level paired analysis further showed that time spent over VT2 (Z3) increased from round 1 to round 2. Seven of the eight athletes showed an increase, one showed no change, and none showed a decrease (Wilcoxon signed-rank test, Z = −2.366, *p* = 0.018).

Given the small sample size, these associations should be interpreted as exploratory, with emphasis placed on the magnitude and direction of the correlations rather than on *p*-values alone.

### 3.4. Mixed-Effects Modeling

A linear mixed-effects model was used to examine changes in mean round heart rate while accounting for repeated observations of athletes (Table 5). The model included 29 valid round-level observations from 8 athletes.

Average round heart rate showed a non-significant increase from Round 1 to Round 2 (β = 3.21 bpm, 95% CI −1.49 to 7.92; *p* = 0.17).

## 4. Discussion

The main aim of this study was to explore how the cardiovascular system of university kickboxers responds during a real KL tournament, identifying the intensity zones according to a previous ventilatory data determination using a progressive treadmill protocol. Overall, the findings show that bouts are predominantly contested at intensities at or above the second ventilatory threshold (VT2), with a clear increase in high-intensity exposure from the first to the second round, and that greater aerobic power is associated with lower internal cardiovascular load during competition.

The ventilatory data indicate that VT1 occurred at approximately half of VO_2_max, whereas VT2 was reached at around 90% of VO_2_max, accompanied by marked increases in ventilatory demand and RER values close to 1.00. This pattern places KL athletes in a physiological profile broadly consistent with that reported in boxing and kickboxing literature, where high VO_2_max and a well-developed anaerobic or ventilatory threshold are repeatedly identified as important contributors to performance [2,3,27].

The literature about VO_2_max determination has reported a variety of methodologies, ranging from different ergometers or equipment (cycle ergometer [5,6]; treadmill [7,28]; step-test [29], to different protocols (multistage shuttle-run test [30]). Additionally, the samples of previous studies differ in terms of training experience (amateur, elite, professional), competitive disciplines (tatami and ring), and gender. All this methodological variability may prevent the comparison of VO_2_max values. The university kickboxers of the present study exhibit a mean value of 52.46 ± 5.14 mL·kg^−1^·min^−1^, which is similar to the values found in a sample of 30 amateur Tunisian kickboxers [5], but lower than the values reported in elite professional Canadian and elite Portuguese kickboxers (above 62 mL·kg^−1^·min^−1^) [5,6], whose samples were composed only of ring sports experienced athletes. A study with 12 males from the Iranian national team (six tatami and six ring-style kickboxers) is the only one, to our knowledge, that presented VO_2_max values of tatami sports athletes [28]. Ring-style kickboxers, as expected, presented a higher value than the tatami kickboxers (52.3 mL·kg^−1^·min^−1^ vs. 47.7 mL·kg^−1^·min^−1^). The repeated high-intensity actions in the sport require well-developed aerobic power [28]. These values may serve as preliminary discipline-specific descriptive data for training prescription and performance monitoring in this population.

Heart rate and intensity-zone data collected during competition reinforce the notion that KL bouts impose substantial physiological demands. In the first round, athletes spent most of the fighting time between VT1 and VT2, with a relevant proportion already above VT2. In contrast, the second round was characterized by a clear shift towards greater time above VT2 and minimal time below VT1. Athlete-level paired analysis confirmed an increase in time spent over VT2 from round 1 to round 2. Seven of the eight athletes showed an increase in Z3, and one showed no change (Z = −2.366, *p* = 0.018). These observations are broadly aligned with evidence from other striking combat sports. De Lira et al. [15] reported that approximately 60% of a simulated Olympic boxing match occurred above VT2, with a progressive increase in high-intensity exposure across rounds. Similarly, Ouergui et al. [8] showed that Light Contact kickboxers spent about 77% of total time in the two highest heart rate zones (80–89% and 90–100% of HRmax), accompanied by elevated blood lactate levels. Although KL was not assessed in those studies, the present findings support the view that tatami disciplines with controlled contact still involve a substantial proportion of time in the upper heavy–severe domain, particularly as bouts progress and tactical intensity accumulates.

Exploratory correlations also suggested some consistency in the relative distribution of intensity between rounds, particularly for Z2 and Z3. This may indicate that athletes tend to reproduce similar relative distributions of effort from the first to the second round, especially at higher intensities. In practice, KL bouts therefore appear to be characterized not by random fluctuations in intensity, but by a gradual intensification of an already high-intensity pattern. Such consistency could be related to deliberate pacing strategies, the cumulative effect of exchanges and fatigue, or a combination of fitness and experience. However, because the HR-zone percentages are compositional and the sample is very small, these correlations should be interpreted descriptively and not as evidence of independent physiological relationships between zones.

The associations between VO_2_max and heart rate responses during the competition provide additional insight into how aerobic power shapes internal load. Higher VO_2_max was strongly associated with a greater proportion of time spent below VT1 in Round 1 and showed a strong inverse association with mean competition heart rate at the athlete level. Specifically, VO_2_max was inversely associated with mean competition heart rate (ρ = −0.719, *p* = 0.045), suggesting that athletes with greater aerobic power tended to experience a lower cardiovascular load during competition. Given the small sample size and exploratory nature of the analysis, this association should be interpreted cautiously. In the context of KL, the current findings reinforce the idea that VO_2_max is not merely a descriptive fitness marker, but a relevant factor in understanding how demanding competition is from a cardiovascular perspective and how close athletes operate to their maximal aerobic limits during official bouts.

The relationship between high-intensity exposure and between-round ΔHR appears more nuanced. At the athlete level, athletes with smaller ΔHR tended to spend more time above VT2 in Round 2 (ρ = −0.690). Although the magnitude and direction of this association may be physiologically relevant, it did not reach statistical significance and should therefore be interpreted cautiously. Beyond combat sports, Lowery et al. [31] demonstrated that VT, and particularly VT2, is more strongly associated with repeated-sprint ability (RSA) than VO_2_peak in competitive ice hockey players and suggested that increasing work rate at VT2 through high-intensity interval training may improve RSA even without changes in VO_2_peak. Together, these findings support a functional link between VT2 adaptations and the ability to sustain and recover from repeated high-intensity efforts, which appears relevant for KL competition. However, the present exploratory findings do not allow firm conclusions regarding the relationship between high-intensity exposure and ΔHR. Further studies with larger samples are needed to determine whether this association is reproducible and independent of other athlete- and competition-level factors.

### 4.1. Limitations and Further Research

Although the present findings provide a preliminary physiological characterization of the cardiovascular behavior of athletes during an official Kick Light competition, several limitations should be considered when interpreting the results. First, the sample was small and drawn from a university Kick Light team (6 men, 2 women), which restricts the generalizability of the findings to other competitive levels, age groups, and kickboxing disciplines. Although subgroup analysis by sex was not feasible given the small sample size, sex-related differences in HR responses, VO_2_max, body composition, and competitive characteristics may have contributed to the observed variability. Therefore, these findings should be viewed as preliminary data from a small, mixed-sex sample rather than as general reference values for Kick Light athletes. The bout structure analyzed (two rounds of two minutes) also reflects a specific university competition format and may differ from other KL or tatami events, as a standard bout consists of three rounds.

Second, heart rate was used as the main index of internal load and between-round recovery. Heart rate is practical, but it is influenced by factors such as pain from impacts, psychological stress, and autonomic regulation, which may not be strictly proportional to metabolic cost. In addition, heart rate does not change instantaneously and may lag behind rapid fluctuations in intensity during intermittent, acyclic activity, such as combat. No complementary measures such as blood lactate, heart rate variability, or detailed time–motion analysis were collected, due to the difficulty in collecting some of those variables in real competition. Therefore, the direct transfer of heart rate zones from a continuous, linear treadmill test to Kick Light competition should be interpreted with caution. The single-day tournament format, in which several athletes competed in more than one bout, may have further influenced heart rate responses and between-round recovery.

Third, the inferential analyses remain constrained by the very small number of independent participants (*n* = 8). Although repeated observations of mean round heart rate were analyzed using a linear mixed-effects model with athlete as a random intercept, the small number of athlete-level clusters limits the precision of both fixed- and random-effect estimates. Model diagnostics also indicated some departure from Gaussianity in the residual tails, although no severe outliers or clear heteroscedasticity were identified. Accordingly, the mixed-model findings should be interpreted as exploratory.

The HR-zone percentages present an additional statistical limitation because Z1, Z2, and Z3 are bound compositional variables that sum to approximately 100% and, in some cases, show substantial floor effects. Gaussian mixed-effects models for these outcomes were therefore not retained. Correlations among the HR-zone percentages are presented only as exploratory descriptions of the observed patterns and should not be interpreted as independent physiological relationships. Likewise, athlete-level correlation analyses were based on only eight observations, resulting in substantial uncertainty even when correlation coefficients were large.

Finally, the cross-sectional design does not allow causal inferences about how changes in VO_2_max, VT1 or VT2 would modify competition behavior or performance. Longitudinal intervention studies in larger and more diverse KL samples are needed to confirm and extend these findings, and they should incorporate complementary physiological markers, such as blood lactate, heart rate variability, or external load, to fully understand the sport’s metabolic demands.

### 4.2. Practical Implications

From an applied perspective, the higher proportion of time spent over VT2, especially in the second round, reinforces the relevance of training strategies that simultaneously develop tolerance to high-intensity effort and the capacity to recover between actions may be relevant for KL athletes. High-Intensity Interval Training (HIIT)-based interventions with strong sport-specific characteristics, integrating intermittent technical–tactical sequences with work–rest structures that replicate competition demands, appear promising for promoting adaptations that increase the VT2 and more efficient heart rate recovery between rounds. However, given the observational and cross-sectional design of this study, causal inferences cannot be drawn, and intervention studies are needed to confirm the effects of specific training. Moreover, the individualized prescription of intensity based on ventilatory thresholds, rather than standardized zones derived from theoretical percentages of HRmax, may optimize the transfer of training to competitive performance and enhance the effectiveness of the preparation process.

## 5. Conclusions

In summary, this study described, for the first time, the cardiovascular responses of university KL athletes during an official competition, relating them to ventilatory thresholds and VO_2_max determined under laboratory conditions. The results indicate a predominance of intensities at or above VT2 through the bout, with increased physiological demands from the first to the second round. Higher VO_2_max was associated with more time spent in lower intensity zones in the early part of the bout and with lower average in-competition heart rate across rounds, suggesting that greater aerobic power was associated with internal cardiovascular load during KL competition. ΔHR was not consistently associated with VO_2_max or time above VT2 in these exploratory analyses, yet the overall pattern indicates that KL imposes substantial intermittent demands that challenge both high-intensity tolerance and ΔHR. These findings provide preliminary, exploratory characterization of heart rate responses during KL competition. Larger studies with more diverse samples are needed to establish discipline-wide reference profiles. Yet, these exploratory findings support the use of individualized metrics to assess and prescribe training, providing meaningful guidance for the design of sport-specific programs aimed at improving performance and recovery in kickboxing KL athletes.

## Figures and Tables

**Table 1:** Anthropometric characteristics of the participants.

Variable	*n* = 8 *
Age (years)	22.50 ± 2.22
Training experience (years)	8.25 ± 7.00
Height (cm)	172.3 ± 6.2
Weight (kg)	71.2 ± 13.0
Fat mass (%)	16.2 ± 6.9

* 6 male, 2 female.

**Table 2:** Descriptive statistics for ventilatory data, for the total sample (*n* = 8).

Variable	Median	M ± SD	Q1	Q3
Ventilatory Threshold 1				
Oxygen consumption (l.min^−1^)	1.95	1.82 ± 0.38	1.48	2.08
Oxygen consumption (ml·kg^−1^·min^−1^)	25.58	26.59 ± 5.33	22.42	31.74
Percentage of VO_2_max	46.10	49.03 ± 8.37	42.30	58.28
Heart rate (bpm)	138.50	137.25 ± 16.78	125.00	151.75
Respiratory exchange ratio (RER)	0.84	0.84 ± 0.08	0.78	0.91
Breathing frequency (Brpm)	28.07	28.93 ± 7.14	22.45	33.34
Ventilatory Threshold 2				
Oxygen consumption (l.min^−1^)	3.29	3.35 ± 0.61	2.95	3.52
Oxygen consumption (ml·kg^−1^·min^−1^)	47.10	46.81 ± 4.33	43.24	50.54
Percentage of VO_2_max	91.62	89.94 ± 6.33	84.17	95.18
Heart rate (bpm)	185.00	183.50 ± 11.33	177.25	192.75
Respiratory exchange ratio (RER)	1.01	1.00 ± 0.04	0.99	1.03
Breathing frequency (Brpm)	42.36	44.34 ± 9.31	37.35	53.36
Maximum Oxygen Consumption (VO_2_max)				
Oxygen consumption (l.min^−1^)	3.73	3.74 ± 0.68	3.15	4.24
Oxygen consumption (ml·kg^−1^·min^−1^)	49.67	52.46 ± 5.14	48.19	58.4
Heart rate (bpm)	194.00	192.38 ± 8.81	183.75	199.75
Respiratory exchange ratio (RER)	1.09	1.08 ± 0.04	1.04	1.11
Breathing frequency (Brpm)	59.24	55.44 ± 9.97	45.38	63.24

**Table 3:** Descriptive statistics for heart rate data, during competitions for the total fights (*n* = 15).

Variable	Median	M ± SD	Q1	Q3
**Round 1**				
total round time (s)	134.00	139.40 ± 16.34	130.00	155.00
%time in Zone 1	0.00	12.03 ± 22.67	0.00	15.70
%time in Zone 2	59.10	52.77 ± 37.72	13.10	96.00
%time in Zone 3	0.70	35.23 ± 42.21	0.00	86.90
average HR	175.00	177.93 ± 8.59	172.00	180.00
final HR	181.00	185.47 ± 8.50	180.00	193.00
rest minimum HR	164.00	161.73 ± 15.85	153.00	167.00
ΔHR	20.00	23.00 ± 9.37	15.00	26.00
**Round 2**				
total round time (s)	125.00	135.00 ± 18.95	121.50	152.25
%time in Zone 1	0.00	3.54 ± 9.01	0.00	0.78
%time in Zone 2	37.75	40.91 ± 35.76	1.50	72.75
%time in Zone 3	46.40	55.55 ± 37.32	27.25	98.50
average HR	181.00	181.50 ± 8.65	179.50	184.50
final HR	186.00	189.14 ± 8.96	183.50	194.50

**Table 4:** Spearman correlation coefficients between VT distribution during competition, aerobic capacity, and heart rate responses (*n* = 8).

	R1%Z1	R1%Z2	R1%Z3	R2%Z1	R2%Z2	R2%Z3	VO_2_max	ΔHR
R1%Z2	0.216							
R1%Z3	−0.559	−0.903 **						
R2%Z1	0.365	0.234	−0.208					
R2%Z2	0.457	0.857 **	−0.927 **	−0.047				
R2%Z3	−0.634	−0.833 **	0.952 **	−0.203	−0.952 **			
VO_2_max	0.913 **	0.000	−0.342	0.483	0.143	−0.381		
ΔHR	0.292	0.292	−0.683	0.514	0.595	−0.690	0.167	
M_HR_COMP	−0.587	−0.311	0.430	−0.768 **	−0.096	0.299	−0.719 *	−0.407

Note: Values are Spearman’s rank correlation coefficients calculated at the athlete level (*n* = 8) after repeated fight observations were averaged within athlete. Because Z1, Z2, and Z3 represent compositional percentages that sum to approximately 100%, correlations among HR-zone variables are presented for descriptive purposes only and were not interpreted as independent physiological associations. No adjustment for multiple comparisons was applied; therefore, all correlations should be considered exploratory. * *p* < 0.05; ** *p* < 0.01.

**Table 5:** Fixed effects from the linear mixed-effects model for mean round heart rate during KL competition.

Outcome	Fixed Effect (Predictor)	Beta (Estimate)	SE	95% CI	*p*-Value
	Intercept (R1)	179.07	2.83	172.71 to 185.42	<0.001
Round_HR_avg_bpm	Round (R2 vs. R1)	3.21	2.26	−1.49 to 7.92	0.170

Note: The model included Round as a fixed effect and Athlete as a random intercept. The model was estimated using restricted maximum likelihood (REML) with Kenward–Roger approximation for denominator degrees of freedom. The analysis included 29 round-level observations from 8 athletes. Random-intercept variance = 42.58 bpm^2^; residual variance = 36.63 bpm^2^; adjusted ICC = 0.538.

## Data Availability

The datasets generated and/or analyzed during the current study are available from the corresponding author on reasonable request.

## Abbreviations

The following abbreviations are used in this manuscript:

BF	Breathing frequency
CI	Confidence interval
ΔHR	Heart rate recovery
HR	Heart rate
HRmax	Maximal heart rate
IOC	International Olympic Committee
KL	Kick Light
RER	Respiratory exchange ratio
RPE	Rating of perceived exertion
SD	Standard deviation
VO_2_	Oxygen uptake
VO_2_max	Maximal oxygen uptake
VT	Ventilatory threshold
VT1	First ventilatory threshold
VT2	Second ventilatory threshold
WAKO	World Association of Kickboxing Organizations
Z1	Zone 1
Z2	Zone 2
Z3	Zone 3
